# CSGALNACT2 restricts ovarian cancer migration and invasion by modulating MAPK/ERK pathway through DUSP1

**DOI:** 10.1007/s13402-023-00903-9

**Published:** 2023-12-12

**Authors:** Mingjun Ma, Chao Wang, Meixuan Wu, Sijia Gu, Jiani Yang, Yue Zhang, Shanshan Cheng, Shilin Xu, Minghai Zhang, Yongsong Wu, Yaqian Zhao, Xiu Tian, Dominic Chih-Cheng Voon, Chiaki Takahashi, Jindan Sheng, Yu Wang

**Affiliations:** 1 Department of Gynecology, Shanghai First Maternity and Infant Hospital, School of Medicine, Tongji University, No.2699, Gaoke West Rd, Shanghai, 200092 China; 2 Shanghai Key Laboratory of Maternal Fetal Medicine, Shanghai Institute of Maternal-Fetal Medicine and Gynecologic Oncology, Clinical and Translational Research Center, Shanghai First Maternity and Infant Hospital, School of Medicine, Tongji University, Shanghai, 200092 China; 3 Department of Obstetrics and Gynecology, Renji Hospital, School of Medicine, Shanghai Jiaotong University, Shanghai, China; 4 Cancer Research Institute, Kanazawa University, Kanazawa, Ishikawa 920-1192 Japan

**Keywords:** Ovarian cancer, CSGALNACT2, Migration and invasion, DUSP1, MAPK/ERK pathway

## Abstract

**Purpose:**

Ovarian cancer is one of the leading causes of cancer-related death among women. CSGALNACT2 is a vital Golgi transferase and is related to a variety of human diseases. However, its expression pattern and function in ovarian cancer remain uncertain.

**Methods:**

The Cancer Genome Atlas and GEPIA databases were used to assess the expression of CSGALNACT2 in ovarian cancer patients. RNA-seq, qRT-PCR, and IHC were used to verify the expression of CSGALNACT2 in ovarian cancer tissues. Then, in vivo and in vitro experiments were conducted to evaluate the role of CSGALNACT2 in the progression of ovarian cancer. RNA-seq and GSEA were used to reveal the potential biological function and oncogenic pathways of CSGALNACT2.

**Results:**

We demonstrated that the mRNA expression and protein level of CSGALNACT2 were significantly downregulated in ovarian cancer and ovarian cancer metastatic tissues. CSGALNACT2 can significantly inhibit the migration, invasion, and clonogenic growth of ovarian cancer in vitro and is progressively lost during ovarian cancer progression in vivo. CSGALNACT2 suppresses ovarian cancer migration and invasion via DUSP1 modulation of the MAPK/ERK pathway through RNA-seq, KEGG analysis, and Western blotting. Moreover, CSGALNACT2 expression was correlated with immune cell infiltration and had prognostic value in different immune cell-enriched or decreased ovarian cancer. In addition, patients with CSGALNACT2 downregulation are less likely to benefit from immunotherapy.

**Conclusion:**

As an ovarian cancer suppressor gene, CSGALNACT2 inhibits the development of ovarian cancer, and it might be used as a prognostic biomarker in patients with ovarian cancer.

**Supplementary Information:**

The online version contains supplementary material available at 10.1007/s13402-023-00903-9.

## Introduction

Epithelial ovarian cancer (EOC) is the most lethal gynecological cancer [1]. As the fifth leading cause of cancer-related deaths in females in the United States, there were an estimated 22,440 new cases of ovarian cancer and 14,080 deaths due to ovarian cancer in 2017 [2]. Most are diagnosed at an advanced stage and will develop recurrent and chemo-resistant disease [3]. Platinum chemotherapy after cytoreductive surgery is still the standard of care in the first-line treatment of ovarian cancer patients. Although most patients initially respond to treatment, the high frequency of drug resistance and recurrence has hindered the success of contemporary anticancer therapies in patients with ovarian cancer, making ovarian cancer the leading cause of death in patients with gynecological malignancies [1]. Various newer therapies, such as anti-angiogenic drugs, PARP inhibitors, and immune checkpoint inhibitors, have become available clinically and have improved survival in patients with ovarian cancer, albeit modestly [4]. Cancer immunotherapy has attracted extensive attention in recent years, and tumor microenvironment (TME) has been proven to play critical roles in anti-tumor immune response [5]. Among the slew of microenvironment factors, the heterogeneity of the immune tumor microenvironment affects the treatment effect of patients and is a potential obstacle to the development of personalized immunotherapy [6]. Therefore, it is urgent to explore novel immune-related predictive biomarkers to provide new hope for improving the prognosis of ovarian cancer patients. Therefore, it is particularly important to further explore the molecular mechanism of the development of ovarian cancer and to actively explore novel immune-related predictive biomarkers to provide new hope for improving the treatment and prognosis of ovarian cancer patients.

Chondroitin sulfate is one of the sulfated glycosaminoglycan (GAG) chains that are covalently attached to various core proteins as proteoglycans, as major components of the connective tissue matrix, and they are also found at the surface of many cell types [7]. In addition, chondroitin sulfate (CS) is one of the common glycosylation types [8], whereas aberrant glycosylation usually regulates the inflammatory response, enables host–pathogen interactions and immune escape, promotes cancer cell metastasis, or regulates apoptosis [9]. Chondroitin sulfate chains have been recognized as critical regulators of growth factor- and cytokine signaling [10–12], and there is ample evidence for a pro-tumorigenic role of chondroitin sulfate in the enhancement of cell proliferation, cell motility, and metastasis [13–15]. Moreover, Chondroitin sulfate N-acetylgalactosami-nyltransferase-2 (CSGALNACT2) is located on chromosome 10q11.22 [7]. It is a vital Golgi transferase that participates in the enzymatic elongation of the chondroitin sulfate chain and initiation of the chondroitin sulfate synthesis [16].

Furthermore, chondroitin sulfate mediates N-cadherin/β-catenin signaling is associated with basal-like breast cancer cell invasion [17], and also controls EGF signaling [18]. In addition, abnormal expression of CSGALNACT2 is associated with osteoarthritic of the knee [19], atherosclerosis [20], pediatric high-grade glioma [21], multiple myeloma [22] as well as colorectal cancer [23]. Meanwhile, the CSGALNACT2 expression was positively associated with the level of immune cells, and high expression of CSGALNACT2 was significantly associated with shorter overall survival (OS) rate in colon adenocarcinoma (COAD) and rectum adenocarcinoma (READ) [23]. However, the mechanism of action of CSGALNACT2 in the occurrence and development of ovarian cancer and its correlation with immune infiltration and treatment of ovarian cancer has not been reported yet.

In this study, we first discovered that CSGALNACT2 was significantly downregulated in ovarian cancer. Subsequently, the role of CSGALNACT2 in the development of ovarian cancer was explored, and its effects and regulatory mechanisms were studied through in vivo and in vitro experiments. As an ovarian cancer suppressor gene, CSGALNACT2 inhibit ovarian cancer migration, and invasion by inhibiting the MAPK/ERK pathway. At the same time, low expression of CSGALNACT2 is associated with the desert immune phenotype of ovarian cancer patients, making it more difficult to benefit from immunotherapy. This research provides a potential target for the pathogenesis and treatment of ovarian cancer.

## Materials and methods

### Patients and specimens

Two groups of ovarian cancer tissues were collected from January 2017. The first group included 28 normal ovarian or fallopian tube tissues, 36 ovarian cancer tissues, and 24 Ovarian cancer metastatic tissues. The second group included 39 ovarian cancer tissues and 16 normal ovarian samples, of which 10 normal ovarian samples and 39 ovarian cancer tissues were used for RNA sequencing, 16 normal ovarian samples, and 16 ovarian cancer tissues were used for qRT-PCR. All patients with ovarian cancer were treated at the Shanghai First Maternity and Infant Hospital. All enrolled patients signed informed consent before surgery, had no history of other malignancies, and had not received chemoradiotherapy. After surgical resection, samples of ovarian cancer tissues or normal tissues were immediately frozen in liquid nitrogen and then transferred to a -80 °C refrigerator for stable storage until use. This study was approved by the Ethics Committee of the Shanghai First Maternity and Infant Hospital. All the included patients signed informed consent forms. The histopathological and clinical data came from pathology reports and medical records.

### Immunohistochemical analysis

Tissue sections were put in 10 mM sodium-citrate buffer and kept at 120℃ for 2.5 min in a pressure cooker to retrieve antigens after dewaxing and rinsing. Then, the tissue sections were blocked in 3% hydrogen peroxide for 15 min in case of intervention of endogenous peroxidase activity. Thereafter, it was incubated with the anti-CSGALNACT2 rabbit polyclonal antibody (1:80, OriGene, USA) at 4 °C overnight and then incubated with the appropriate secondary antibodies for 1 h at room temperature and stained with DAB and hematoxylin. Lastly, the tissue sections were covered with coverslips for microscopic observation. Staining intensity was scored on the following scale: 0 (negative staining), 1 (weak staining), 2 (moderate staining), and 3 (strong staining). The proportion of positively stained areas was evaluated with five levels: 0 (< 5%), 1 (5%–25%), 2 (25%–50%), 3 (50%–75%), and 4 (> 75%). The final score was the product of the above two indicators.

### Cell lines and cell culture

The primary cells were derived from the ovarian primary and metastases of ovarian cancer patients who received surgical treatment for the first time in our hospital, and the extraction and culture methods were described [24]. The human ovarian cancer lines HEY, OVCAR8, A2780, and normal ovarian epithelial cell line IOSE were purchased from the American Type Culture Collection (ATCC, Manassas, VA). HEY and A2780 were maintained in Dulbecco’s modified Eagle’s medium (DMEM medium; Gibco, USA), and RPMI-1640 medium (Gibco, USA) was used to cultivate OVCAR8, IOSE cells with 10% fetal bovine serum (FBS, Gibco) and 1% penicillin–streptomycin, in a humidified atmosphere of 5% CO2 at 37 °C.

All cell lines were regularly authenticated according to the recommendations of the ATCC cell bank using short tandem repeat polymorphism analysis. All cell lines were mycoplasma-free.

### RNA extraction and quantitative real-time PCR

Total RNA was extracted from cell lines by using TRIzol reagent (Takara, Japan) according to the instructions of the manufacturer. The cDNA was synthesized with random primers using an ABScript III RT Master Mix for qPCR with gDNA remover (Abclonal, Wuhan, China); BrightCycle Universal SYBR Green qPCR Mix with UDG (Abclonal, Wuhan, China) was used to perform a qRT-PCR reaction according to the guidelines of the manufacturer. GAPDH was used as an internal control. TRIzol reagent was used to extract total RNA from the tissue or cell line according to the manufacturer’s instructions. β-Actin and GAPDH were used as internal controls. The 2 − ΔΔCT method was used to analyze relative expression levels. The primers were all synthesized by Sangon Biotech (Shanghai, China). Primer sequences are:

- *GAPDH* forward: GAAGGTGAAGGTCGGAGTC
- *GAPDH* reverse: GAAGATGGTGATGGGATTTC
- *β-Actin* forward: CATGTACGTTGCTATCCAGGC
- *β-Actin* reverse: CTCCTTAATGTCACGCACGAT
- *CSGALNACT2* forward:AACAAAGAGCAAGCACCTAGTG
- *CSGALNACT2* reverse: TGGCCCCTATGCTAACTTCAG
- *CDC25A* forward: GAATGAGGAGGAGACCCCCT
- *CDC25A* reverse: TGGCAAGCGTGTCATTGTTG
- *RASSF6* forward: ACAGGACCCAGATTCCTATGTC
- *RASSF6* reverse: GCTGCTTCACTCATGGTTCTAT
- *DUSP1* forward: ACCACCACCGTGTTCAACTTC
- *DUSP1* reverse: TGGGAGAGGTCGTAATGGGG
- *TXNIP* forward: ATATGGGTGTGTAGACTACTGGG
- *TXNIP* reverse: GACATCCACCAGATCCACTACT

### RNA sample processing and sequencing

HEY cells were transfected with the empty vector control or oeCSGALNACT2 plasmids. Total RNA was extracted from cell lines by using TRIzol reagent (Takara, Japan) according to the instructions of the manufacturer. The extracted RNA samples were checked for overall quality, and only samples with high quality (RIN ≥ 8.0) and high purity (OD 260/280 = 1.8–2.0) were used to perform RNA-seq by Novogene (Tianjin, China). Statistical analysis and visualization of gene sets were performed using the clusterProfiler R package. The RNA-seq data presented in this study were submitted to the Gene-Expression Omnibus.

### Western blotting

One percent PMSF added to RIPA lysis buffer (Beyotime, China) was used to extract total proteins. Lysates containing equal amounts of protein were loaded onto each lane, followed by separation using 10% SDS polyacrylamide gel electrophoresis and transferred onto a PVDF membrane (Millipore, Billerica, MA, USA) at 300 mA for 100 min. The membranes were blocked at room temperature for 1 h in 5% skim milk solution and subsequently incubated with primary antibodies at 4 °C overnight and then with the secondary antibody at room temperature for 1 h and visualized using electrochemiluminescence. β-Actin was the internal control. CSGALNACT2 antibody (1:5000, Abcam, #ab181250), PAX8 antibody (1:2000, Proteintech, #10,336-1AP), β-Actin antibody (1:10,000, Proteintech, #81,115–1-RR), GAPDH antibody (1:20,000, Proteintech, #60,004–1-Ig), ERK1/2 antibody (1:1000, Abmart, # T40071), Phospho-ERK1/2 antibody (1:2000, Proteintech, # 28733–1-AP).

### Plasmid constructs, cell transfection, and virus infection assay

For the construction of overexpression plasmids. Firstly, after amplifying the target fragment through PCR, the vectors were cut using restriction endonucleases, and then T4 ligase were used to connect the two, allowing full-length human CSGALNACT2 to be cloned into the pLVX-IRES-Puro vector. Finally, the target fragment was transferred to the host bacteria and the recombinant clone was obtained through screening and identification.

For the construction of knockdown plasmids, the CSGALNACT2 small hairpin RNA (shRNA) plasmids were designed through the Thermo Fisher Scientific website, and synthesized by General biol (Anhui, China). The primer was first annealed, followed by pLKO.1-puro vector digestion at 37° for 2 h. Then, the annealed primers were connected to the digested vector at 16° C overnight using T4 ligase. Finally, the target fragment was transferred to the host bacteria and the recombinant clone was obtained through screening and identification.

For lentivirus preparation, 293 T cells were transfected with plasmid DNA using Lipofectamine 3000 (Invitrogen, Carlsbad, CA, USA) according to the instructions of the manufacturer. The medium contained lentivirus was collected after 48 h, centrifuged at 3000 rpm for 5 min, and then the supernatant was collected. Cells were infected with a medium containing lentivirus and supplemented with 8 μg/ml polybrene by Genomeditech (Shanghai, China) for 48 h. After successful transfection, cell lines were screened with puromycin for 30 days. The above oligonucleotide sequences are:

- *OE-CSGALNACT2*forward: ATGCCTAGAAGAGGACTGATTCTTCAC
- *OE-CSGALNACT2*reverse: AATCCACCAATGGTCAGGAAATCTGA
- *shCSGALNACT2#1* forward: GCATAGGCTATCAGAGCAACA
- *shCSGALNACT2#1* reverse: TGTTGCTCTGATAGCCTATGC
- *shCSGALNACT2#3* forward: GCTTGGAGGTCATTAATAATC
- *shCSGALNACT2#3* reverse: GATTATTAATGACCTCCAAGC

### Cell wound-healing assay and transwell assays

For the cell wound-healing assay, 2 × 10^5^ (HEY), 2.5 × 10^5^ (OVCAR8) cells were cultured in a 6-well plate to 90% confluence, respectively. A 200-µl pipette tip was used to create a scratch of the same width. Then, the cells were incubated in a serum-free medium, and images were captured at 0 and 48 h after injury to evaluate the migration rate. All experiments were repeated independently in triplicate.

For Transwell assay, 1 × 10^5^ (A2780), 2 × 10^4^ (HEY), 5 × 10^4^ (OVCAR8),8 × 10^4^ (IOSE) cells of each group in 200 µl serum-free medium were seeded in the upper chamber (#3422, Corning, USA) without (migration) or with (invasion) Matrigel (#356,234, BD Bioscience, USA), respectively. 500 µl culture medium with 10% FBS was added to the lower chamber. After incubating for 24 h, the upper chambers were fixed with 4% paraformaldehyde for 15 min and then stained with 0.5% crystal violet for 15 min. Then, cotton swabs were used to remove the cells inside the upper chamber. A microscope was used to take pictures and then evaluate the number of cells with ImageJ software. Five random fields were selected to calculate cells of migration and invasion.

### Cell counting Kit‑8 (CCK‑8) assay and clonogenic assay

The Cell proliferation assay was performed using Cell counting kit-8 (CCK8, Dojindo, Japan) following the manufacturer's guidelines. Briefly, 1.5 × 10^3^ (HEY), 2 × 10^3^ (OVCAR8), 2.5 × 10^3^ (A2780), 2 × 10^3^ (IOSE) cells were seeded at an appropriate density in 96-well plates and cultured at 37 °C, respectively. After culturing for 0, 24, 48, 72, and 96 h, CCK-8 solution (10 µL) was added to each well, incubated for 2 h in an incubator, and then a spectrophotometer was used to evaluate the absorbance at 450 nm.

For the clonogenic assay, 1000 cells were seeded in 6-well plates at 800 cells per well in triplicate wells. After two to three weeks of culture, the cells were fixed with 4% paraformaldehyde for 30 min and stained with 0.5% crystal violet solution for 30 min. and colonies > 1 mm were counted using ImageJ.

### 5‑ethynyl‑20‑deoxyuridine (EdU) assay

The EdU detection assay was performed using a Cell-Light EdU DNA cell proliferation kit (#CX003, Beyotime Biotechnology, China) following the manufacturer's guidelines. 2 × 10^4^ (HEY), 2.5 × 10^4^ (OVCAR8), 3 × 10^4^ (A2780), 2.5 × 10^4^ (IOSE) cells were incubated with EdU for 2 h and then fixed with 4% paraformaldehyde, followed by dyeing and sealing with Apollo dye solution and Hoechst 33,342. Finally, an inverted fluorescence microscope (Carl Zeiss, Germany) was used to take pictures to evaluate the proportion of EdU-positive cells.

### In vivo studies

All animal care and experiments were conducted according to the guidelines of the National Institutes of Health and approved by the Animal Care Committee of Tongji University. Six-to-6-week-old female C57BL/6 mice were anesthetized with isoflurane and a single dorsal incision was made to access to ovary. ID8 cells (2.0 × 10^6^) were then injected into the bilateral ovarian bursa. After 10 days, 20 days, and 30 days, respectively, the mice were euthanized, and the primary ovarian lesion, Colon, omentum, and mesenteric metastases were dissected, embedded in paraffin, and finally validated by hematoxylin and eosin (H&E) staining, then perform immunohistochemistry.

### Statistical analysis

Statistical analysis was performed using GraphPad Prism software (Version 8.0.2, GraphPad Software, Inc., USA). Student’s t-test (two-tailed) was used to compare the differences between the two groups, and one-way ANOVA was used to compare the differences among multiple groups. Pearson correlation analysis was used to analyze the correlations. Kaplan–Meier analysis was performed to compare the overall survival (OS) rate between the high and low CSGALNACT2 gene expression groups using the p-value determined in the log-rank test. All data are presented as the means ± SD. In all analyses, *P* < 0.05 was considered statistically significant. *, *P* ≤ 0.05; **, *P* ≤ 0.01; ***, *P* ≤ 0.001; ****, *P* ≤ 0.0001; no asterisks, P not calculated.

## Results

### CSGALNACT2 was down-regulated and associated with better prognosis in ovarian cancer

**Fig. 1:**
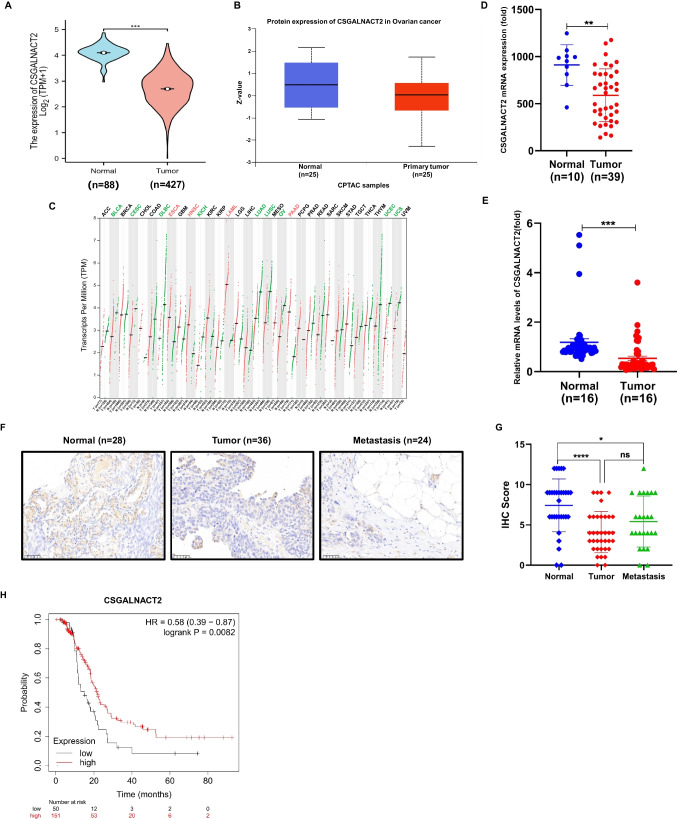
CSGALNACT2 was down-regulated and associated with a better prognosis in ovarian cancer. **A** The expression of CSGALNACT2 in TCGA-OV from the UALCAN database (https://ualcan.path.uab.edu/). **B** The protein level of CSGALNACT2 in CPTAC from the UALCAN database. **C** CSGALNACT2 gene expression level from GEPIA2.0 (http://gepia2.cancer-pku). It includes 426 TCGA-OV samples and 88 GTEx normal samples. **D** RNA-seq was used to detect relative expressions of CSGALNACT2 in 10 normal and 39 tumor tissues of ovarian cancer. **E** qRT-PCR was performed to detect relative levels of CSGALNACT2 in normal and tumor tissues (*n* = 16) of ovarian cancer. **F** The protein level of CSGALNACT2 in normal ovarian epithelial tissues, ovarian cancer, and ovarian cancer metastatic tissues detected by immunohistochemical (IHC) staining. All micrographs (× 40; scale bar, 50 mm) were imaged from one representative case. **G** IHC scores between normal ovarian epithelial tissues, ovarian cancer, and ovarian cancer metastatic tissues. **H** Kaplan–Meier analysis of CSGALNACT2 on progression-free-survival (PFS) of ovarian cancer patients with Paclitaxel treatment from Kaplan–Meier plotter (https://kmplot.com/). *p ≤ 0.05, ***p ≤ 0.001

To determine the clinical significance of CSGALNACT2 in ovarian cancer, we assessed the expression of CSGALNACT2 in ovarian cancer patients by analyzing a public database. As shown in Fig. 1A and B, the results showed that the expression of CSGALNACT2 was lower in ovarian cancer compared with normal samples. Additionally, CSGALNACT2 was also decreased in a variety of cancer types, such as bladder Urothelial Carcinoma (BLCA), cervical squamous cell carcinoma and endocervical adenocarcinoma (CESC), lymphoid Neoplasm Diffuse Large B-cell Lymphoma (DLBC), kidney Chromophobe (KICH), Uterine Corpus Endometrial Carcinoma (UCEC) (Fig. 1C and Supplementary Fig. 1A).

Next, we further conducted RNA-seq to explore the expression level of CSGALNACT2 in 10 normal and 39 ovarian cancer tissues. The results showed that CSGALNACT2 was significantly higher in normal tissues (Fig. 1D), and the qRT-PCR experimental results also confirmed that CSGALNACT2 is noteworthy upregulated in normal ovarian tissue (Fig. 1E). Moreover, we detected the protein level of CSGALNACT2 using ovarian cancer tissues, which contained 28 normal and 36 tumor tissues, and 24 ovarian cancer tissues with metastasis. Normal ovarian epithelial samples exhibited stronger staining of CSGALNACT2 than both ovarian cancer and ovarian cancer metastatic tissues (Fig. 1F and G). And elevated CSGALNACT2 expression had longer survival outcomes in ovarian cancer patients with Paclitaxel or Docetaxel treatment by Kaplan–Meier survival analysis (Fig. 1H, Supplementary Fig. 1B and C).

### CSGALNACT2 inhibited the migration, invasion, and clonogenicity of normal ovarian epithelial cell line

**Fig. 2:**
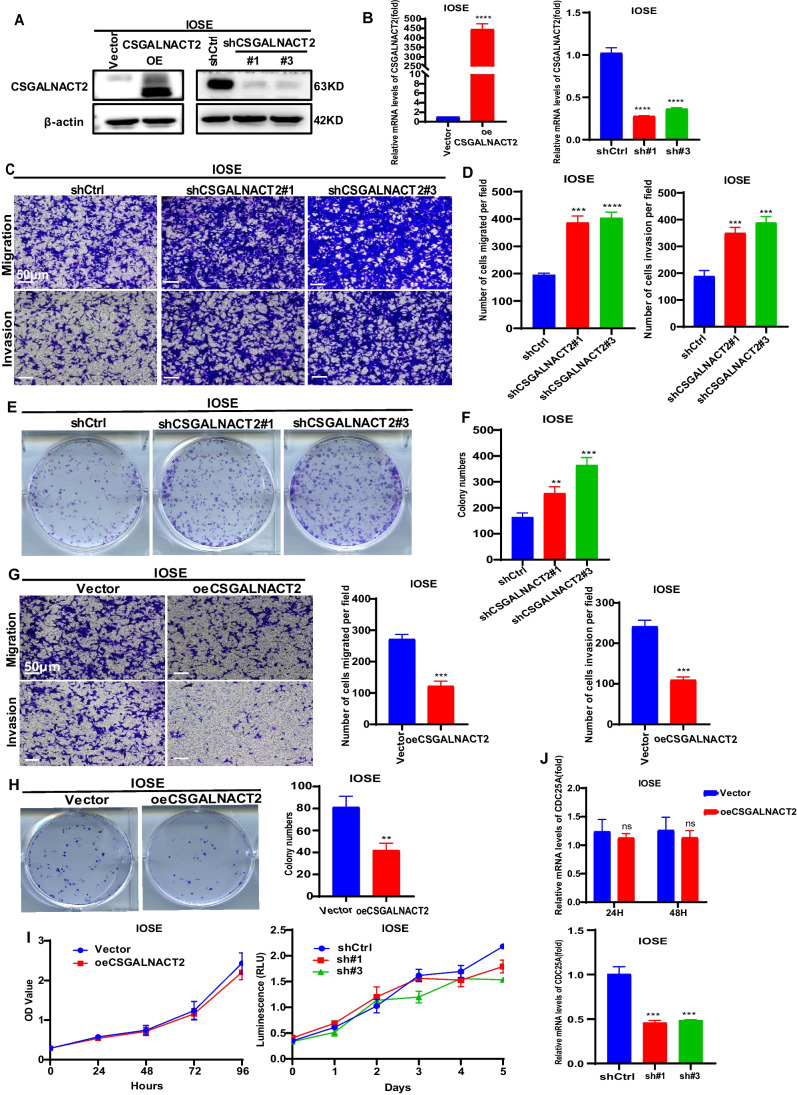
CSGALNACT2 inhibited the migration, invasion, and clonogenicity of normal Ovarian epithelial cell lines. **A-B** CSGALNACT2 overexpression and knockdown efficiency validation in IOSE cells by Western blotting and qRT-PCR. **C-D** Transwell assays detected the migration and invasion ability of cells after the knockdown of CSGALNACT2. Scale bar = 50 μm. **E–F** Stable knockdown of CSGALNACT2 promoted clonogenic growth in IOSE cell lines. **G** Transwell assays detected the migration and invasion ability of cells after overexpression of CSGALNACT2. Scale bar = 50 μm. **H** Colony formation assays were used to detect the proliferation ability of cells after overexpression of CSGALNACT2. **I-J** CCK-8 assays and the CDC25A expression were used to detect changes in cell proliferation after overexpression or knockdown of CSGALNACT2 in IOSE cells. **, *P* ≤ 0.01; ***, *P* ≤ 0.001; ****, *P* ≤ 0.0001; ns, not statistically

Based on the above results, we first explored the effects of CSGALNACT2 on normal ovarian epithelium. We constructed IOSE cell lines with stable knockdown or overexpression of CSGALNACT2 (Fig. 2A and B) and determined the effect of CSGALNACT2 on cell migration and invasion using the transwell assays.. We detected that CSGALNACT2 knockdown markedly promoted migration and invasive activities in IOSE cells (Fig. 2C and D). The clone formation assay also showed that IOSE cells formed more numerous and larger cell colonies after CSGALNACT2 expression was reduced (Fig. 2E and F). On the contrary, overexpression of the CSGALNACT2 significantly inhibited the migration and invasion of IOSE cells, as well as the ability of clonogenicity (Fig. 2G and H). Interestingly, overexpression or knockdown of the CSGALNACT2 gene in normal ovarian epithelial cells did not have a significant impact on cell proliferation (Fig. 2I, J and Supplementary Fig. 2A, B). These results indicate that the reduced expression of CSGALNACT2 could contribute to the migration and invasion of transformed ovarian epithelial cells.

### CSGALNACT2 suppressed complex oncogenic phenotypes of ovarian cancer cells

**Fig. 3:**
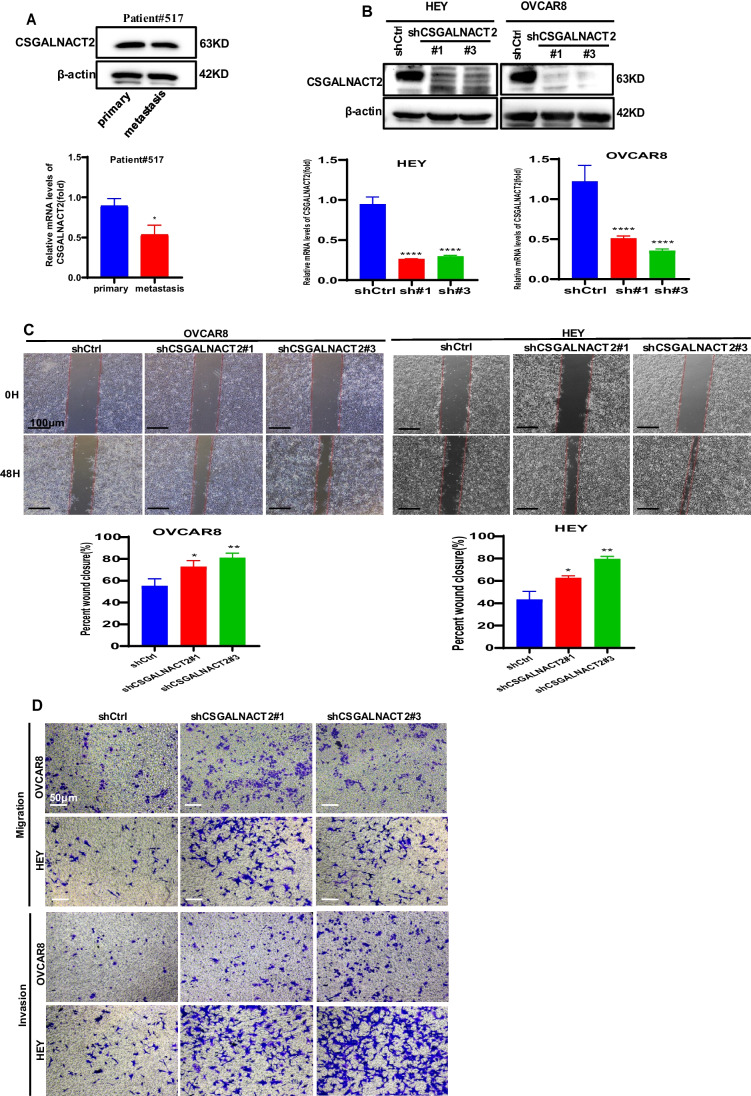

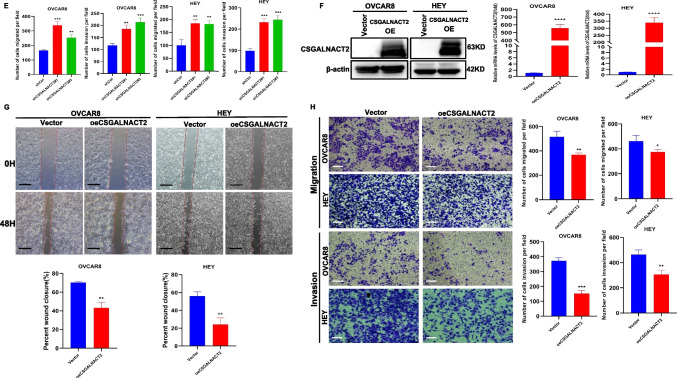

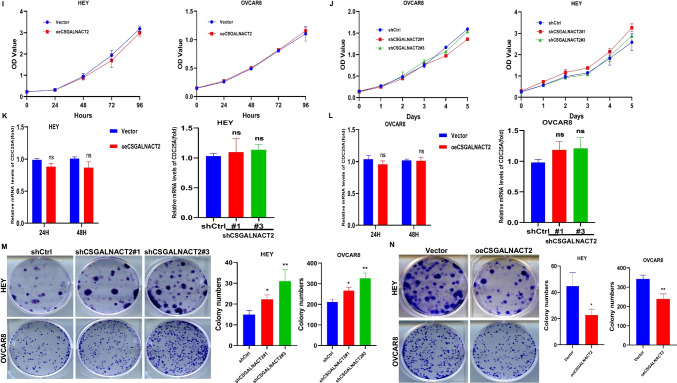

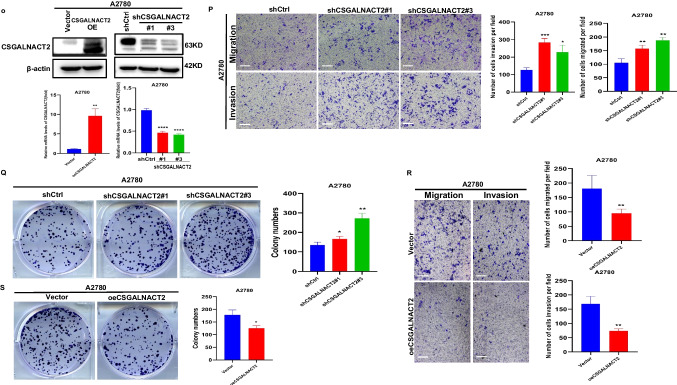
CSGALNACT2 is required for the suppression of cell mobility, metastasis, and clonogenic growth in ovarian cancer cells. **A** Protein and mRNA expressions of CSGALNACT2 in primary and metastatic cell lines of ovarian cancer patients were analyzed by Western Blotting and qRT-PCR. The expression of β-actin is an internal loading control. **B** Knockdown efficiencies of CSGALNACT2 in HEY and OVCAR8 cells were analyzed by Western blotting and qRT-PCR. shCtrl: cells infected with scrambled shRNA virus; shCSGALNACT2: cells infected with shCSGALNACT2 virus. **C** Wound-healing assays after CSGALNACT2 knockdown by shRNA in OVCAR8 and HEY cells. Scale bar = 100 μm. **D-E** Effects of CSGALNACT2 inhibition on cancer cell migration and invasion for OVCAR8 and HEY cells were analyzed; scale bar = 50 μm. And quantification of migrated and invaded cells of CSGALNACT2-silenced cells was presented as mean ± S.D. **F** Protein and mRNA expression of CSGALNACT2 in CSGALNACT2-overexpressed HEY and OVCAR8 cells was examined by Western blotting and qRT-PCR. **G** Wound-healing assays after CSGALNACT2 overexpression in OVCAR8 and HEY cells. Scale bar = 100 μm. **H** The transwell migration and invasion assays of CSGALNACT2 overexpression in OVCAR8 and HEY cells were determined and quantified. Results presented as the mean ± S.D. Scale bar = 50 μm. **I-J** CCK-8 assays were used to detect changes in cell proliferation after overexpression or knockdown of CSGALNACT2 in OVCAR8 and HEY cells. **K-L** The expression of proliferation marker CDC25A in cell proliferation after overexpression or knockdown of CSGALNACT2 in OVCAR8 and HEY cells by qRT-PCR. **M–N** Colony formation assays were used to detect the proliferation ability of cells after overexpression or knockdown of CSGALNACT2 in OVCAR8 and HEY cells. **O** CSGALNACT2 overexpression and knockdown efficiency validation in A2780 cells by Western blotting and qRT-PCR. **P** Transwell assays after CSGALNACT2 knockdown in A2780 cells. Scale bar = 50 μm. **Q** Colony formation assays were used to detect the proliferation ability of cells after the knockdown of CSGALNACT2. **R-S** Transwell and colony formation assays were used to detect the migration, invasion, and proliferation ability of A2780 cells after overexpression or knockdown of CSGALNACT2. Scale bar = 50 μm. *, *P* ≤ 0.05; **, *P* ≤ 0.01; ***, *P* ≤ 0.001; ****, *P* ≤ 0.0001; no asterisks, *P* not calculated

High-grade serous ovarian carcinoma (HGSOC) is the most common form of ovarian cancer, accounting for over 70% of cases and the majority of deaths. The observation of CSGALNACT2 downregulation in ovarian cancer tissue and metastatic lesions encourages us to further investigate the impact of experimental downregulation of CSGALNACT2 on HGSOC in vitro. First of all, we examined the expression of CSGALNACT2 in primary cells from both primary and metastatic ovarian lesions of the same ovarian cancer patient in our hospital. Interestingly, the basal expression level of CSGALNACT2 was lower in metastasis cells than in primary cells (Fig. 3A). To further determine the role of CSGALNACT2, we used two different shRNA constructs targeting the CSGALNACT2 transcript to knockdown CSGALNACT2 expression in two HGSOC cell lines, HEY and OVCAR8, according to the mRNA and protein expression of CSGALNACT2 in normal ovarian epithelial cell line (IOSE) and ovarian cancer cell lines (Supplementary Fig. 3A). The efficiencies of transfection were validated by qRT-PCR and Western blotting (Fig. 3B). We first determined the role of CSGALNACT2 on cell migration and invasion using the wound-healing and transwell assay. The results showed that the downregulation of CSGALNACT2 significantly enhanced ovarian cancer cell migration and invasion abilities (Fig. 3C-E). In contrast, overexpression of CSGALNACT2 dramatically inhibited the migration and invasion abilities in both HEY and OVCAR8 cells (Fig. 3F-H), implying that CSGALNACT2 may suppress HGSOC cell mobility. Next, we evaluated the functional impact of CSGALNACT2 on HGSOC cell proliferation using the CCK8 and EdU assays and detected the expression of cell proliferation marker CDC25A. As shown in Fig. 3I-L and Supplementary Fig. 3B-C, CSGALNACT2-depleted or overexpressed cells and control cells exhibited similar growth kinetics. These observations indicate that CSGALNACT2 in HGSOC cells does not lead to significant changes in cell proliferation ability. Next, we assessed whether CSGALNACT2 knockdown promotes clonogenic growth. Both HEY and OVCAR8 cells expressing the control nontargeting shRNA were modestly clonogenic, respectively. However, CSGALNACT2 depletion in both cell lines significantly increased their clonogenicity (Fig. 3M). In contrast, overexpression of CSGALNACT2 inhibited clonogenicity in HGSOC cells (Fig. 3N).

To further investigate the impact of changes in CSGALNACT2 gene expression on other ovarian cancer cell lines, two shRNA-targeted CSGALNACT2 and overexpression plasmids of CSGALNACT2 were designed and transfected into A2780 cell (Fig. 3O). Next, we evaluated the impact of CSGALNACT2 downregulation on proliferation, clonogenic, and cell migration and invasion. We observed that downregulation of CSGALNACT2 significantly enhanced A2780 cell migration, invasion, and clonogenicity abilities (Fig. 3P and Q), whereas overexpression of CSGALNACT2 obtained opposite results (Fig. 3R and S). However, as expected, CSGALNACT2 did not significantly affect the proliferation of A2780 cells (Supplementary Fig. 3D and E). The above results indicate that CSGALNACT2 not only plays an inhibitory role in ovarian high-grade serous cancer cells but also has an inhibitory effect in ovarian endometrioid adenocarcinoma cells.

### CSGALNACT2 expression is reduced during the metastasis of ovarian cancer in vivo

**Fig. 4:**
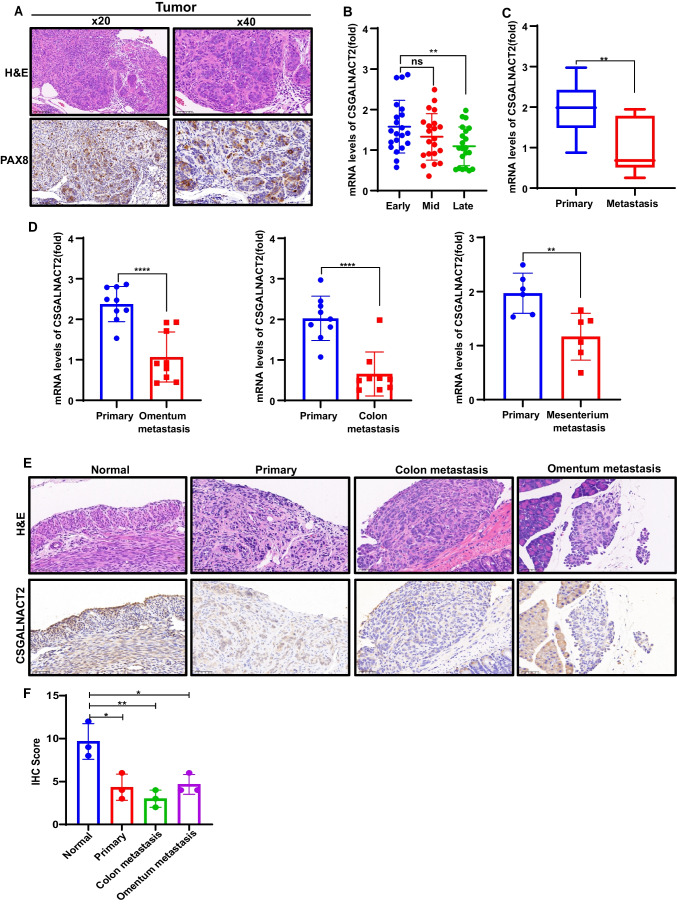
CSGALNACT2 expression is reduced during the metastasis of ovarian cancer in vivo. **A** H&E staining (top) and Immunohistochemistry (IHC) (bottom) of tissues. Immunohistochemistry (IHC) using specific antibodies targeting PAX8 (IHC Score = 12). The scale bar represents 100 μm (Left panels). The scale bar represents 50 μm (Right panels). **B** The mRNA expression levels of CSGALNACT2 in different stages of ovarian cancer primary by qRT-PCR. **C-D** The expression of CSGALNACT2 in ovarian primary and different metastatic lesions was detected by qRT-PCR. **E–F** H&E staining (top) and Immunohistochemistry (IHC) (bottom) of tissues. The expression levels of CSGALNACT2 in primary ovarian lesions and different metastatic lesions of each group were detected by immunohistochemical staining. Scale bar = 50 μm

To evaluate the potential of CSGALNACT2 on metastasis of ovarian cancer in vivo, we constructed an ovarian in situ implantation model using injection with ID8 cells into the ovary of C57BL/6 mice. According to the preliminary experimental results, ascites and widespread metastasis in the abdominal cavity appeared in the mice at 1 month (Supplementary Fig. 5). Therefore, we divided 15 mice into three groups, each consisting of five mice. One mouse was maintained normally, while other four mice made bilateral ovarian in situ tumors. Then, the mice were euthanized on day 10 (Early), day 20 (Mid), and day 30 (Late) to analyze the expression of CSGALNACT2 at different stages (Supplementary Fig. 5). Firstly, we performed H&E and immunohistochemical staining on the cancer tissue, exhibiting stronger staining of PAX8, indicating that the tumor is serous cancer (Fig. 4A). And, we observed that the expression of CSGALNACT2 decreased gradually as the tumor progressed, and the expression was significantly lower in the late stage than in the early (Fig. 4B). At the same time, the expression of CSGALNACT2 in tumor metastasis was significantly lower than that in the primary lesion (Fig. 4C). Specifically, the expression of CSGALNACT2 was significantly reduced in omental metastases, colonic metastases, and mesenteric metastases (Fig. 4D). In addition, IHC staining analysis showed that CSGALNACT2 expression levels were lower in ovarian cancer tissues and metastatic tissues in the middle and late stages (Fig. 4E and F). These observations reveal a negative correlation between the expression of CSGALNACT2 and the progression and metastasis of ovarian cancer in our vivo model. Although these data do not explicitly demonstrate a suppressive role for CSGALNACT2 in preventing ovarian metastasis, they implicate CSGALNACT2 to serve an impeding function that should be further investigated in future studies.

### CSGALNACT2 suppresses ovarian cancer migration and invasion via DUSP1 modulation of MAPK/ERK pathway

**Fig. 5:**
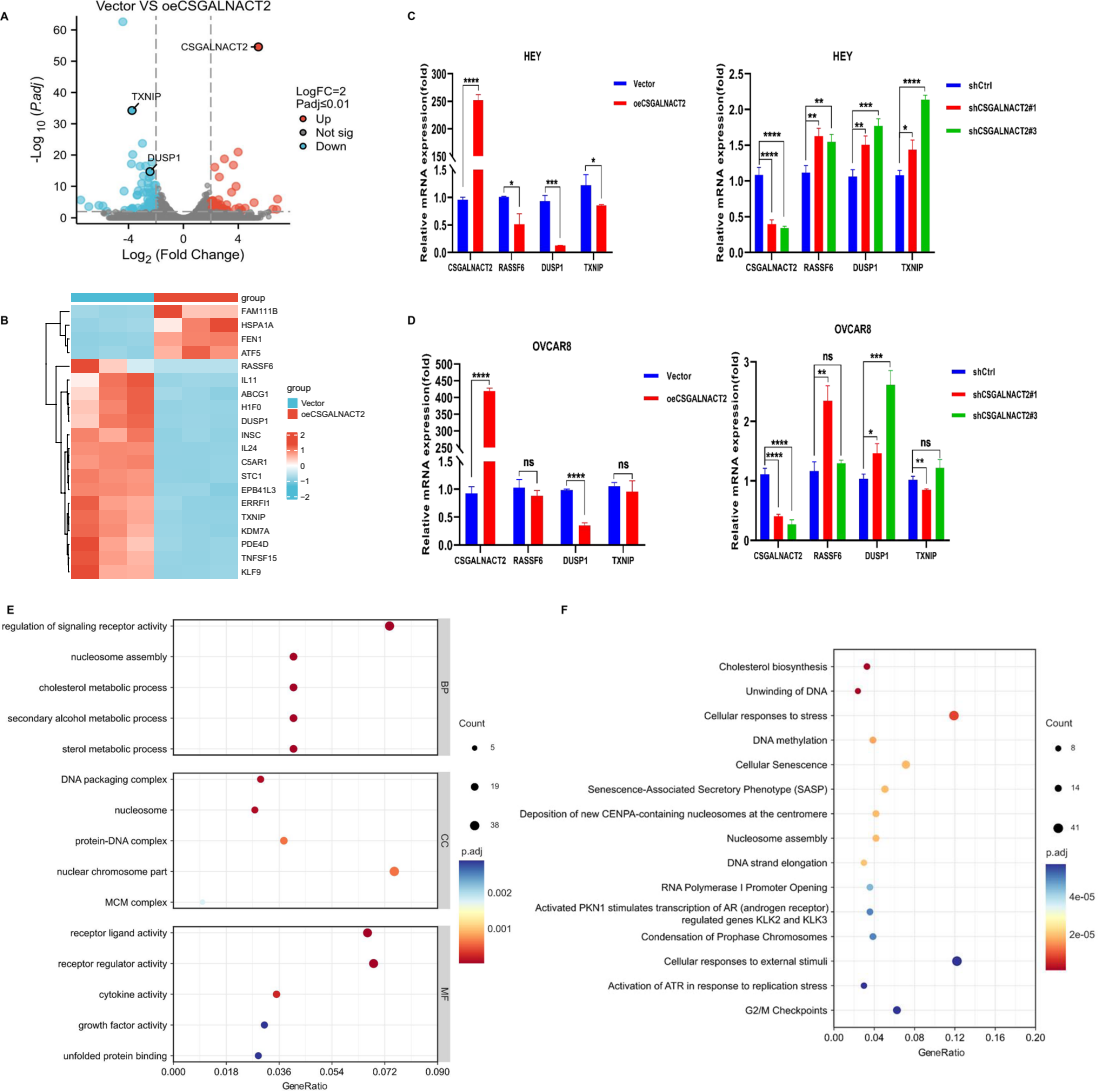

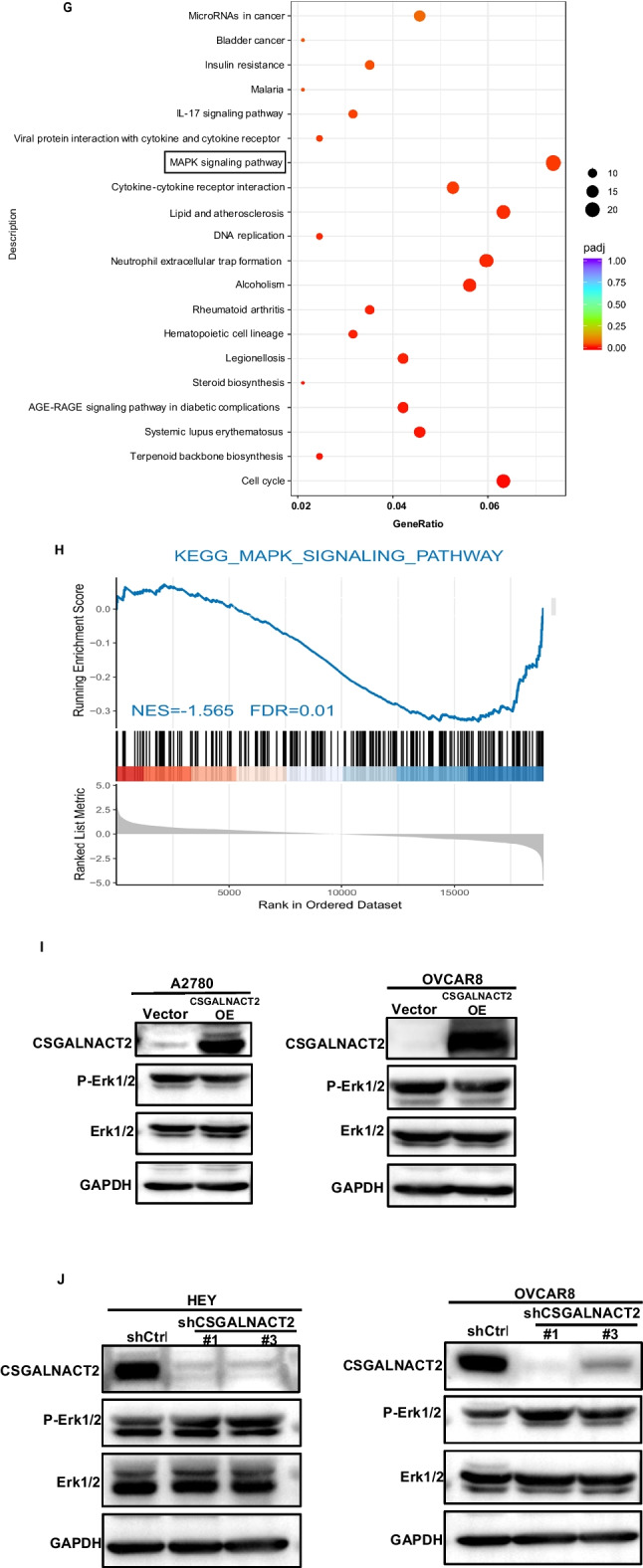
CSGALNACT2 suppresses ovarian cancer migration and invasion via DUSP1 modulation of MAPK/ERK pathway. **A** Volcano plot of the distribution of differentially expressed genes (DEGs) by RNA-seq in HEY cell lines with stable overexpression of CSGALNACT2. Red and blue dots represented statistically significant up- and down-regulated CSGALNACT2, respectively. **B** Heatmap showed the 20 DEGs with the most significant up- and down-regulation in the upregulated CSGALNACT2 group compared with the control group. **C-D** qRT-PCR analyzed the expression of core genes, such as RASSF6, DUSP1, TXNIP in HEY cells **C** and OVCAR8 cells **D** with up- and down-regulated CSGALNACT2. *, *P* ≤ 0.05; **, *P* ≤ 0.01; ***, *P* ≤ 0.001; ****, *P* ≤ 0.0001; ns, not statistically. **E** The correlation of GO biological process, molecular function, and CSGALNACT2 was analyzed by GSEA via RNA-Seq data. **F** Reactome analysis of the relationship between various reactions and biological pathways and CSGALNACT2 expression via RNA-Seq data. **G-H** The correlation of KEGG pathways and CSGALNACT2 was analyzed by GSEA via RNA-Seq data. **I-J** MAPK signaling pathway related proteins (p-Erk1/2, Erk1/2) were detected by western blotting in HEY, OVCAR8 and A2780 cells

To further explore the potential molecular mechanisms underlying the role of CSGALNACT2 in the development of ovarian cancer, we conducted RNA sequencing and analyzed mRNA differentially regulated mRNAs of HEY cells transfected with Vector and oeCSGALNACT2. The volcano map results showed that 273 genes were up-regulated and 343 genes were down-regulated after CSGALNACT2 overexpression (Fig. 5A). The heat map showed that the expression of the top 20 genes with the most significant differences when CSGALNACT2 was upregulated, RASSF6, DUSP1, and TNXIP expression was strongly downregulated (Fig. 5B). This was further confirmed by qRT-PCR in HEY, OVCAR8, A2780, and IOSE cells, and the difference in DUSP1 expression was the most significant (Fig. 5C, D and Supplementary Fig. 4A, B). These results suggest that CSGALNACT2 may inhibit the motility and clonogenic ability of ovarian cancer cells through downregulated of DUSP1 expression.

To explore the potential biological process and molecular function of CSGALNACT2 in ovarian cancer, we used GSEA to analyze the correlation of CSGALNACT2 expression with biological processes and molecular functions. The enrichment results of Gene Ontology (GO) enrichment analysis are shown in Fig. 5E. There existed statistically significant differences in terms of the enrichment score of receptor regulator or ligand activity, nuclear chromosome part, and regulation of signaling receptor activity. And, the results of Reactome analysis indicated that cellular responses to external stimuli and cellular responses to stress were associated with CSGALNACT2 expression (Fig. 5F). Moreover, GSEA analysis was also performed to explore the correlation between CSGALNACT2 expression and oncogenic pathways. KEGG pathway analysis demonstrated that the expression of CSGALNACT2 in ovarian cancer was correlated with the MAPK signaling pathway, Lipid and atherosclerosis, and Cell cycle, and the highest correlation was with the MAPK signaling pathway (Fig. 5G and H, Supplementary Table 1). Furthermore, we investigated the activity of MAPK/ERK pathway by measuring ERK1/2 phosphorylation levels following the RNAi knockdown or over-expression of CSGALNACT2. The over-expression of CSGALNACT2 resulted in a slight reduction of p-ERK1/2 in A2780 and OVCAR8 cell lines (Fig. 5I). To investigate if this modest increase was due to the sufficiency of endogenous CSGALNACT2, we conducted RNAi knockdown of endogenous CSGALNACT2 in OVCAR8 cells and observed significant increases in p-ERK1/2 levels (Fig. 5J). A similar trend was observed in HEY cells in which endogenous CSGALNACT2 expression was appreciable (Fig. 5J). Together, these data provide additional evidence that CSGALNACT2 exerts its effects via negatively regulating a subset of MAPK activity, likely downstream of DUSP1.

### Correlation analysis of CSGALNACT2 with immunotherapy and immune cell infiltration

**Fig. 6:**
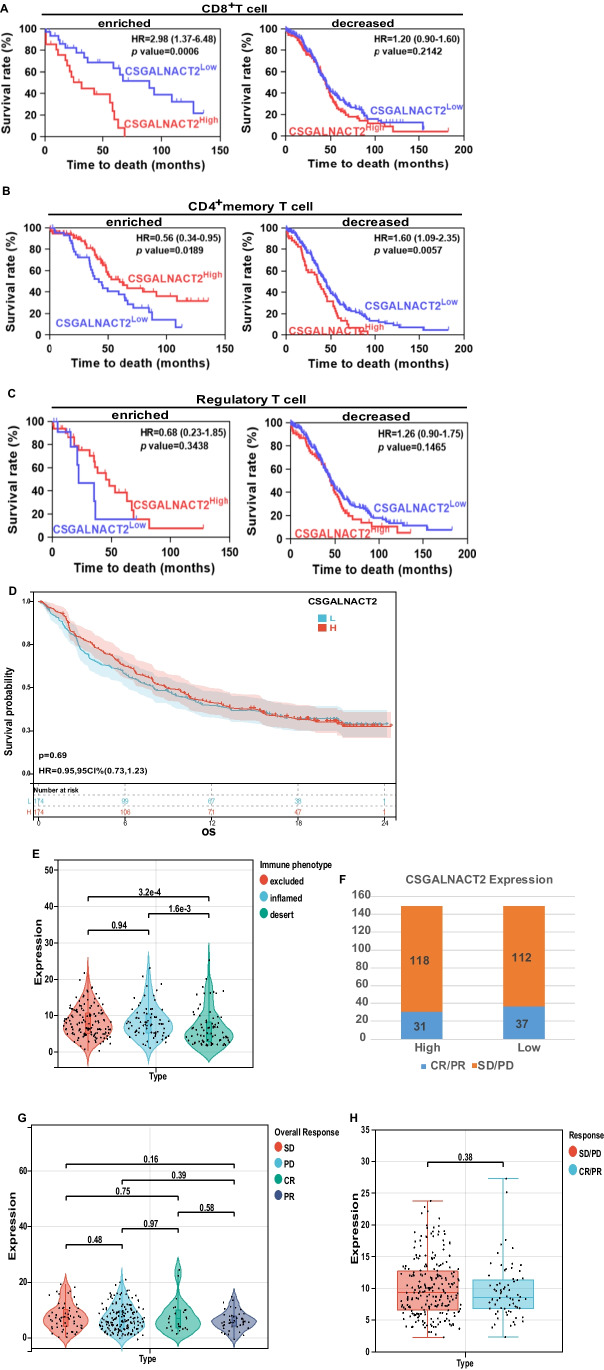
Correlation analysis of CSGALNACT2 with immunotherapy and immune cell infiltration. **A** Kaplan–Meier curves were plotted based on the different groups of CSGALNACT2/CD8 + T cell enriched or decreased, CSGALNACT2/CD4 + memory T cell enriched or decreased, **B** CSGALNACT2/Regulatory T cell enriched or decreased **C** in TCGA cohort. **D** Survival analyses for high (174 cases) and low (174 cases) CSGALNACT2 expression patient groups in the anti-PD-L1 immunotherapy cohort using Kaplan–Meier curves (IMvigor210 cohort). **E** Differences in CSGALNACT2 among distinct tumor immune phenotypes in the IMvigor210 cohort. The lines in the boxes represented the median value by the Kruskal–Wallis test. **F** The number of patients with response to PD-L1 blockade immunotherapy in high or low CSGALNACT2 expression groups. SD, stable disease; PD, progressive disease; CR, complete response; PR, partial response. **G** The distribution of CSGALNACT2 in different anti-PD-L1 clinical response groups. **H** The correlation of CSGALNACT2 with immunotherapy response, Wilcoxon rank sum was applied for the significance test

Previous studies have confirmed that the abnormal number of different immune cells could affect the tumor's immune response and CSGALNACT2 expression was positively associated with the level of immune cells in colon adenocarcinoma (COAD) and rectum adenocarcinoma (READ) [23]. To further investigate the effect of CSGALNACT2 expression on ovarian cancer prognosis under different immune cell-enriched or decreased conditions, we performed a survival analysis of ovarian cancer patients in the TCGA cohort. Here, Kaplan–Meier analysis showed that the CD8^+^T cell enriched/ CSGALNACT2high group was associated with worse prognosis of ovarian cancer patients, compared with the CD8^+^T cell enriched/ CSGALNACT2low group (HR = 2.98, *P* = 0.0006). While, when CD8^+^T cells decreased, the abnormal expression of CSGALNACT2 did not affect the prognosis of ovarian cancer (Fig. 6A). However, the CD4^+^T cell enriched/ CSGALNACT2high group was associated with a better prognosis of ovarian cancer patients, but the CD4^+^T cell decreased / CSGALNACT2high group predicted poor survival of ovarian cancer patients (Fig. 6B). In addition, the expression of CSGALNACT2 did not affect the prognosis of patients with ovarian cancer regardless of the increase or decrease of regulatory T cells (Fig. 6C).

Prompted by the distinct correlation between CSGALNACT2 expression and patient prognosis in the context of specific immune cell composition, our interest was drawn to the potential role of CSGALNACT2 in influencing the clinical outcome of ICB therapies, such as anti-PD-L1 immunotherapy. To this end, we interrogated an immunotherapeutic dataset of urothelial cancer called IMvigor210 cohort from Mariathasan et al. [25]. From the results, we could see that CSGALNACT2 upregulation had no significant impact on survival (Fig. 6D). In addition, the correlation of different CSGALNACT2 expression with different immune phenotypes (desert, excluded, inflamed) was also explored, the results indicated that patients with desert immune phenotype were more likely to unfavorable for immunotherapy, while the other two types exhibited the opposite (Fig. 6E). The results demonstrated that higher CSGALNACT2 expression was found to be associated with inflamed and excluded immune phenotypes while the lower one was associated with the desert type, which meant that patients with CSGALNACT2 downregulation are less likely to benefit from immunotherapy. In addition, we further investigated the therapeutic responsiveness and clinical benefits of anti-PD-L1 immunotherapy by comparing different CSGALNACT2 expression cohorts, we found that the expression of CSGALNACT2 had no significant impact on immunotherapy in cancer patients (Fig. 6F-H). The above results strongly indicated that CSGALNACT2 could represent the immune status of TME, which might play an essential part in forecasting the response to immunotherapy as well.

## Discussion

Ovarian cancer is one of the most lethal gynecological malignancies [26]. One of the main factors contributing to the high mortality rate is the advanced stage of the disease at diagnosis. The 5-year relative survival rate for late-stage disease was 29%, compared to 92% for early-stage disease [27]. Although surgical resection active chemotherapy and radiation therapy have been proven to be beneficial, the prognosis of ovarian cancer remains poor [28]. There has been no substantial improvement in survival rates for ovarian cancer patients over the past two decades [29]. These adverse clinical outcomes are largely a result of unresolved metastases, relapses, and the occurrence of intrinsic or acquired drug resistance [30]. Therefore, a better understanding of the underlying pathways of tumorigenesis, progression, and metastasis may help to design more effective treatment strategies for ovarian cancer.

Several studies have investigated the role of chondroitin sulfate in cancer, providing evidence for its important role in cell proliferation, migration, apoptosis, and differentiation [31, 32]. Chondroitin sulfate N-acetylgalactosami-nyltransferase-2 is a vital Golgi transferase that participates in the enzymatic elongation of the chondroitin sulfate chain and initiation of the chondroitin sulfate synthesis [16]. Previous studies have revealed that CSGALNACT2 is associated with osteoarthritic of the knee, atherosclerosis, pediatric high-grade glioma, multiple myeloma, as well as colorectal cancer [19–23]. The high expression of CSGALNACT2 was significantly associated with a shorter Overall survival rate in COAD and READ [23]. Martinez-Romero et al. [33] have shown that upregulation of CSGALNACT2 is associated with poor prognosis in colorectal cancer. However, unlike previous studies, this study found that CSGALNACT2 expression is downregulated in ovarian cancer and ovarian cancer metastasis, and high expression is associated with better prognosis in ovarian cancer patients.

There are no studies that have evaluated the role of CSGALNACT2 in the regulation of ovarian cancer cells in ovarian cancer. Based on this phenomenon, we explored the possible role and mechanism of CSGALNACT2 in ovarian cancer. The results of functional experiments showed that CSGALNACT2 can inhibit migration, invasion, and clone formation of ovarian cancer while promoting the motility of normal ovarian epithelium. Research showed that CSGALNACT2, as well as CSGALNACT1, exhibited GalNAcT activity in both the initiation and elongation of the CS synthesis in vitro [16]. Decreased expression of CSGALNACT1 can increase the numbers of dead and apoptotic cells and significantly decrease cell viability in prostate cancer cells [34]. And CSGALNACT2 deletion attenuated aortic smooth muscle cell migration [35]. Different from previous studies, our study showed that CSGALNACT2 overexpression inhibited migration and invasion, as well as the ability to clone normal ovarian epithelium, while CSGALNACT2 knockdown inhibited these functions. At the same time, we showed that downregulation of CSGALNACT2 promoted clonogenic growth and motility in normal ovarian epithelial cells (IOSE) with no effect on cell proliferation. The observed experimental phenomena are in agreement with a previous report by Jagmohan Hooda and colleagues [36], who reported a stable loss of H2Bub1-promoted clonogenic growth and motility in immortalized FTSECs with little to no effect on cell proliferation. Clonogenic growth and enhanced cell migration are distinctive features of advanced tumors, suggesting that decreased CSGALNACT2 expression in ovarian neoplasms may potentially facilitate cancer progression. Moreover, the fact that CSGALNACT2 suppressed clonogenicity without impacting cellular proliferation suggests that it acts specifically on a subset of MAPK-mediated function pertaining to migration and colonization downstream of DUSP1.In IHC analysis of clinical normal ovarian tissue, ovarian cancer tissue, and metastatic tissue, the expression of CSGALNACT2 gradually decreased and significantly decreased in metastatic lesions, which was confirmed by qRT-PCR and RNA-seq. Meanwhile, IHC analysis of in vivo experiments again showed that CSGALNACT2 expression gradually decreased with the progression of ovarian cancer, with the lowest expression in advanced stages and metastases.

Previous studies have shown that chondroitin sulfate in the ECM acts on tumor cells to promote tumor progression. chondroitin sulfate can mediate N-cadherin/β-catenin signaling [17] and also can control EGF signaling [18]. It is well known that the typical MAPK (mitogen-activated protein kinase) signaling pathway regulates a range of tumorigenesis [37], and its activity is self-limiting under physiological conditions, dephosphorylation and inactivation of the MAPK signaling pathway through rapid phosphorylation and upstream inhibition, such as DUSPs [38]. However, the pathway can be continuously activated under abnormal conditions, thus driving tumor progression [39]. Dual-specificity phosphatase 1 (Dusp1), also known as MAPK phosphatase 1, is a nuclear mitogen and stress-inducible MAPK phosphatase [40], which can negatively modulate the MAPK pathway by dephosphorylation of MAPKs, and participate in various cellular responses, including inflammation, cell proliferation, and apoptosis [41]. In this study, RNA-seq analysis of the stable strain after CSGALNACT2 overexpression showed that DUSP1 was significantly down-regulated, when stable depletion of CSGALNACT2, DUSP1 expression was significantly increased. At the same time, KEGG enrichment analysis of RNA-seq results showed that the MAPK pathway was significantly enriched. Furthermore, we confirmed that overexpression of CSGALNACT2 resulted in a slight reduction of p-ERK1/2 in A2780 and OVCAR8 cell lines and knockdown of CSGALNACT2 can significantly increase p-ERK1/2 levels in OVCAR8 and HEY cells. To sum up, this study for the first time found that CSGALNACT2 exert its biological function through the MAPK/ERK pathway.

Recently, researchers have had a strong interest in the field of immunotherapy, which has been proven to be effective in the treatment of human malignant tumors [42]. It is reported that the phenotype and function of major immune cell subsets (including bone marrow cells, macrophages, dendritic cells, and T cells) in the ovarian cancer microenvironment have changed in response to immunotherapy [43]. Mounting studies have indicated that several immune infiltrating cells are conducive to the favorable prognosis of patients with ovarian cancer, especially CD8^+^T cells, CD4^+^T cells, and natural killer cells [44–46]. Previous research has demonstrated that the CSGALNACT2 expression was positively associated with the level of immune cells (including dendritic cell, neutrophil, macrophage, CD4^+^T Cell, and CD8^+^T Cell), and high expression of CSGALNACT2 was significantly associated with shorter overall survival (OS) rate in COAD and READ [23]. In this study, we observed that the expression of CSGALNACT2 is correlated with immune cells (including CD8^+^T cells, CD4^+^T cells, and regulatory T cells). The result showed that the CD8^+^T cell enriched/ CSGALNACT2high group was associated with a worse prognosis of ovarian cancer patients. However, the CD4^+^T cell enriched/ CSGALNACT2high group was associated with a better prognosis of ovarian cancer patients. In addition, the expression of CSGALNACT2 did not affect the prognosis of patients with ovarian cancer regardless of the increase or decrease of regulatory T cells. Currently, immunotherapy, especially immune checkpoint blocking (ICB) therapy, has achieved impressive success in cancers such as melanoma and non-small cell lung cancer [47], but the therapeutic value of immunotherapy in OC is still under investigation [48]. According to the status of tumor-infiltrating immune cells (TICs) in TME, tumors can be divided into two different types: hot tumors and cold tumors [49]. Although the tumor mutation burden (TMB) of OC is relatively high, it still falls into the category of cold tumors. Therefore, the overall response rate of immunotherapy for ovarian cancer is still unsatisfactory. Fangfang Xu and colleagues [50] have proven that higher expression of APOBEC3A was found to be strongly associated with inflamed immune phenotype, while the lower one was associated with the excluded and desert types, and higher APOBEC3A expression was tightly related to a better clinical response to immunotherapy through an immune cohort study. Interestingly, this study demonstrated that higher CSGALNACT2 expression was associated with inflamed and excluded immune phenotypes while the lower one was associated with the desert type, which meant that patients with CSGALNACT2 downregulation are less likely to benefit from immunotherapy. The above results strongly indicated that CSGALNACT2 could represent the immune status of TME, which might play an essential part in forecasting the response to immunotherapy as well. However, how CSGALNACT2 regulates immune infiltration and its role in anti-tumor immune response remains to be further studied.

## Conclusions

In summary, our research suggests that CSGALNACT2, as a new tumor suppressor gene in ovarian cancer, inhibit the development of ovarian cancer through the MAPK/ERK pathway. Meanwhile, CSGALNACT2 is associated with different immune cell infiltration, and low expression of CSGALNACT2 in ovarian cancer patients is not conducive to immunosuppressive therapy. Our findings provide a new potential target for the development and treatment of ovarian cancer.

### Supplementary Information

Below is the link to the electronic supplementary material.

Supplementary file1 (DOCX 20585 kb)

Supplementary file2 (XLS 43 kb)

Supplementary file3 (XLSX 13 kb)

## Data Availability

The data are available within the Article, Supplementary Information, or available from the authors upon request. Source data are provided with this paper.
