# Supplementary material for: CSGALNACT2 restricts ovarian cancer migration and invasion by modulating MAPK/ERK pathway through DUSP1
Source: Cell Oncol (Dordr). 2023 Dec 12;47(3):897–915. doi: 10.1007/s13402-023-00903-9 (PMC11219422; doi:10.1007/s13402-023-00903-9)
Supplement: Supplementary file 1 — Supplementary file1 (DOCX 20585 kb) [file 13402_2023_903_MOESM1_ESM.docx]

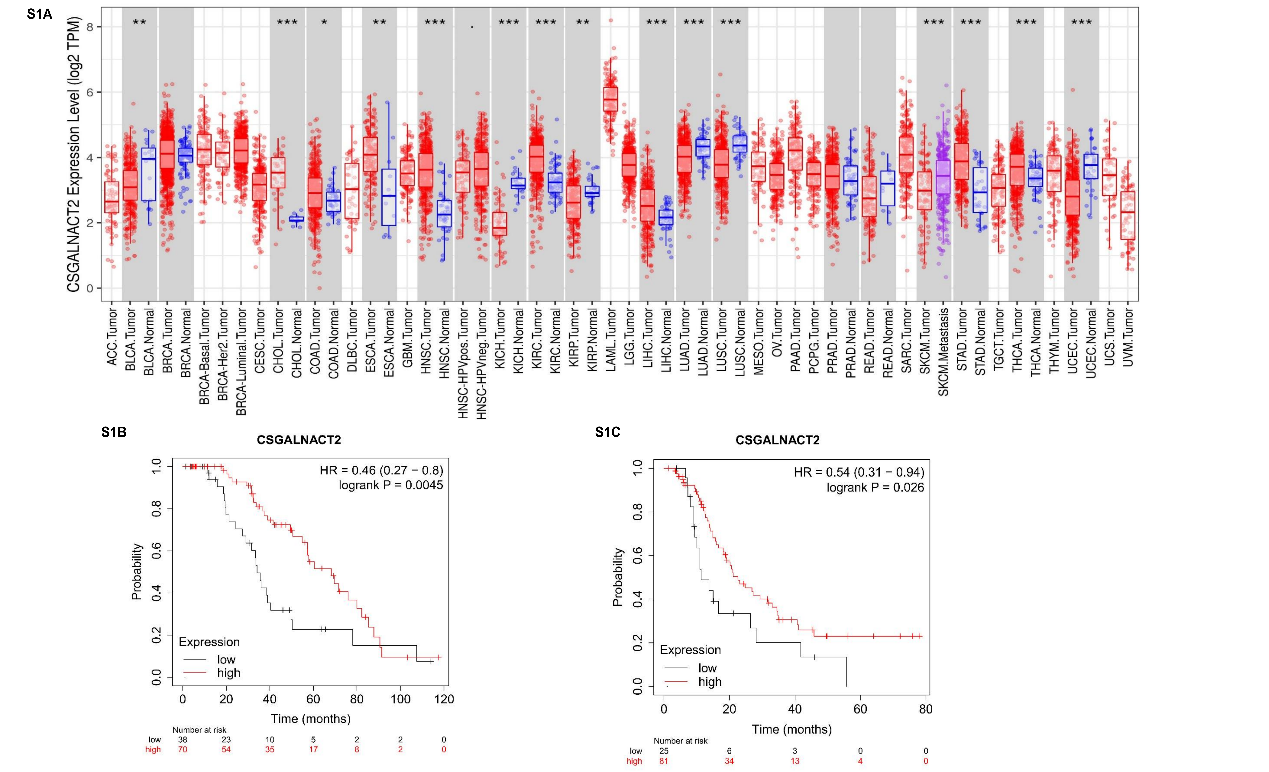


**Supplementary** **Fig. 1** **A** The differential expression between tumor and adjacent normal tissues for CSGALNACT2 across different types of cancers via the TIMER 2.0 ( [http://timer.cistrome.org/](%20http://timer.cistrome.org/)). **B-C** Kaplan–Meier analysis of CSGALNACT2 on overall survival (OS) and progression-free survival (PFS) of ovarian cancer patients with Docetaxel treatment from Kaplan–Meier plotter. *p < 0.05, ** p < 0.01, ***p < 0.001.


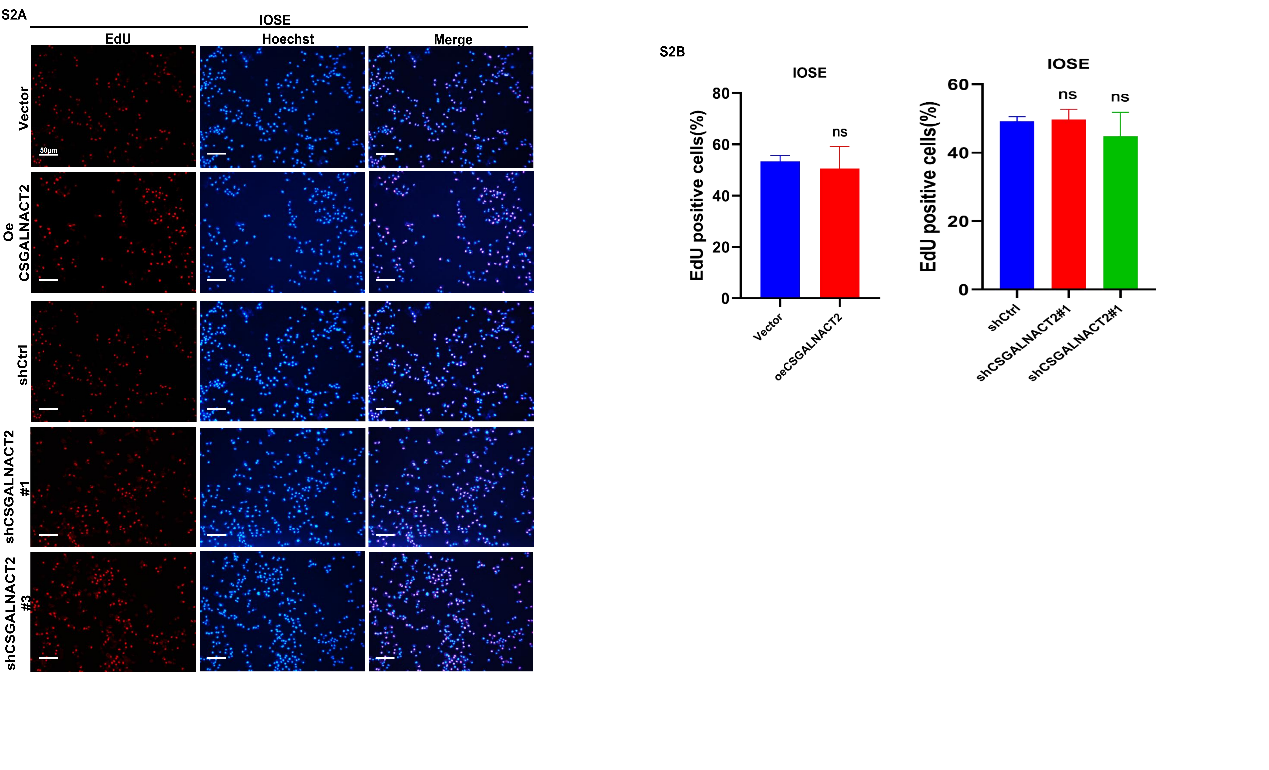


**Supplementary** **Fig. 2** **A-B** EdU assays were used to find out changes in cell proliferation after overexpression or knockdown of CSGALNACT2 in IOSE cells. Scale bar=50μm. ns, not statistically.


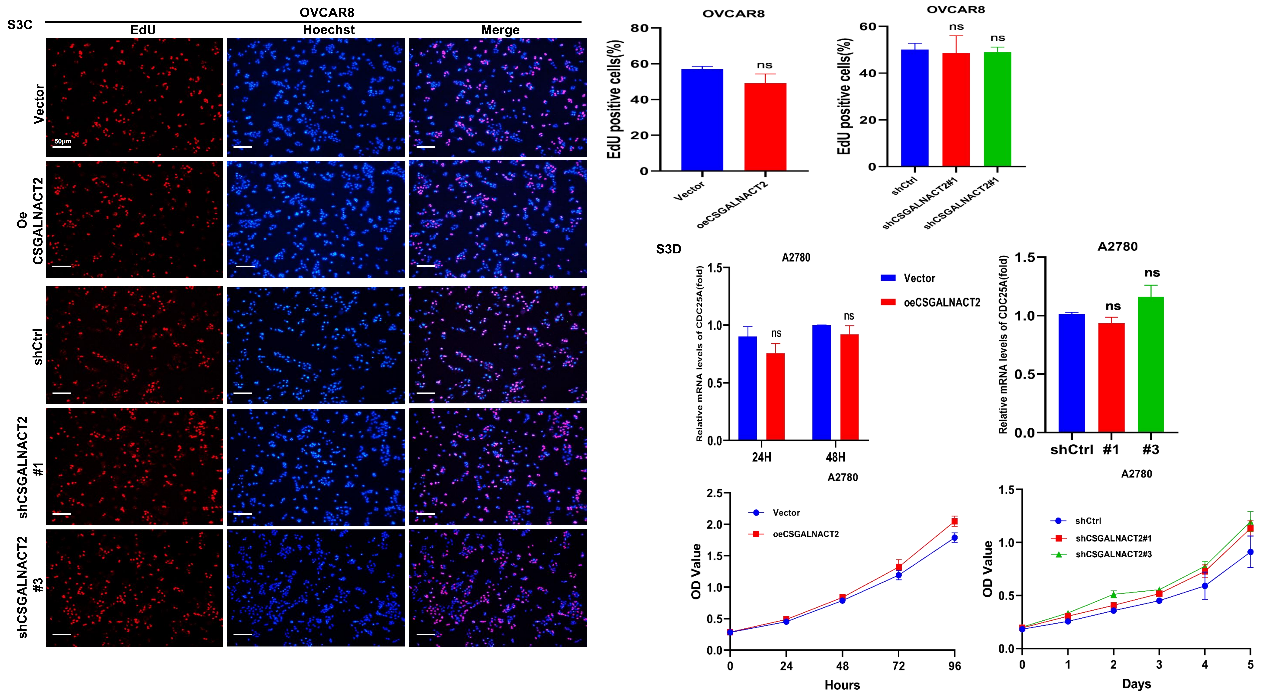

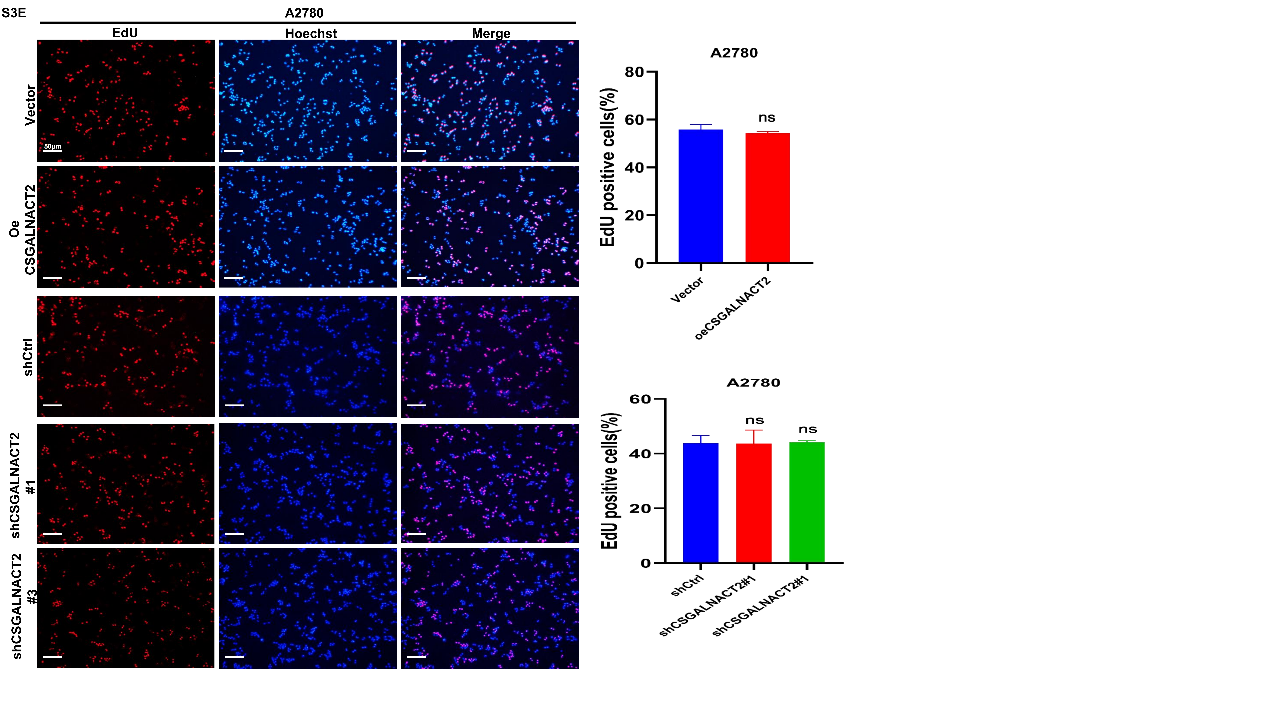

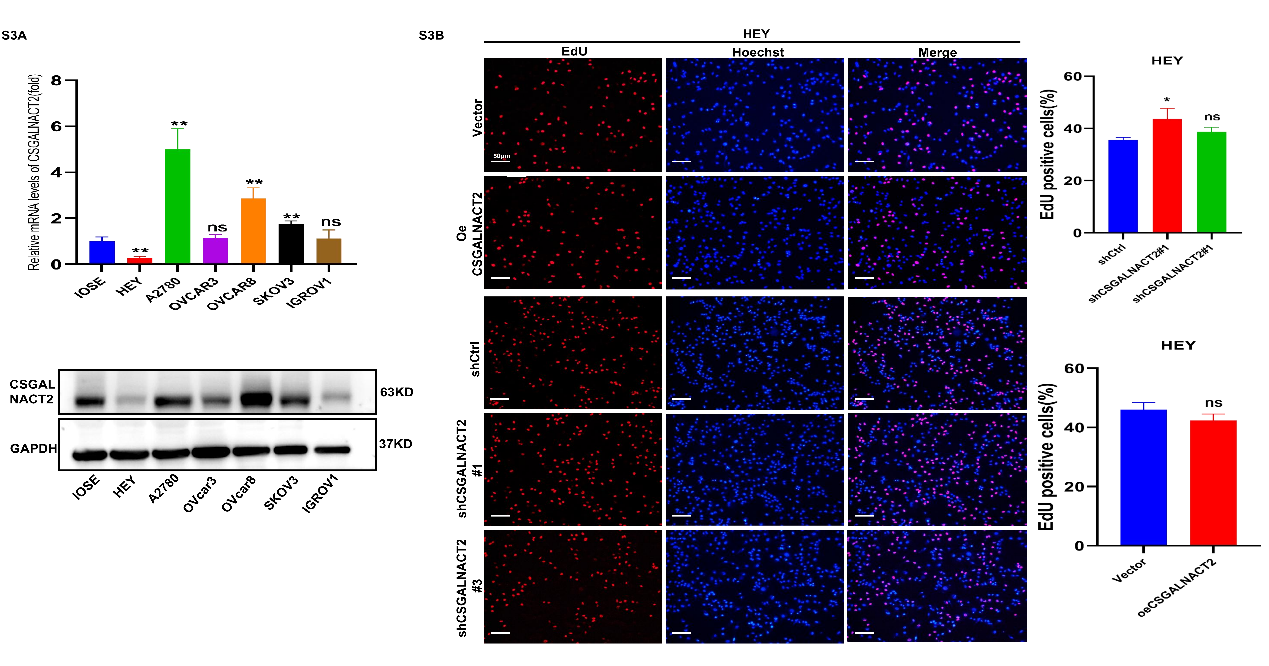


**Supplementary** **Fig. 3** **A** qRT-PCR and Western Blotting detected CSGALNACT2 mRNA and protein expression in normal ovarian epithelial cell line IOSE and six ovarian cancer cell lines. Original blots/gels are presented in Supplementary Figure 6F. **B-C** EdU assays were used to detect changes in cell proliferation after overexpression or knockdown of CSGALNACT2 in OVCAR8 and HEY cells. Scale bar=50μm. **D-E** CCK-8 assays and EdU assays were used to detect changes in cell proliferation after overexpression or knockdown of CSGALNACT2 in A2780 cells. Scale bar=50μm. *p≤0.05, ** p≤0.01, ns, not statistically.


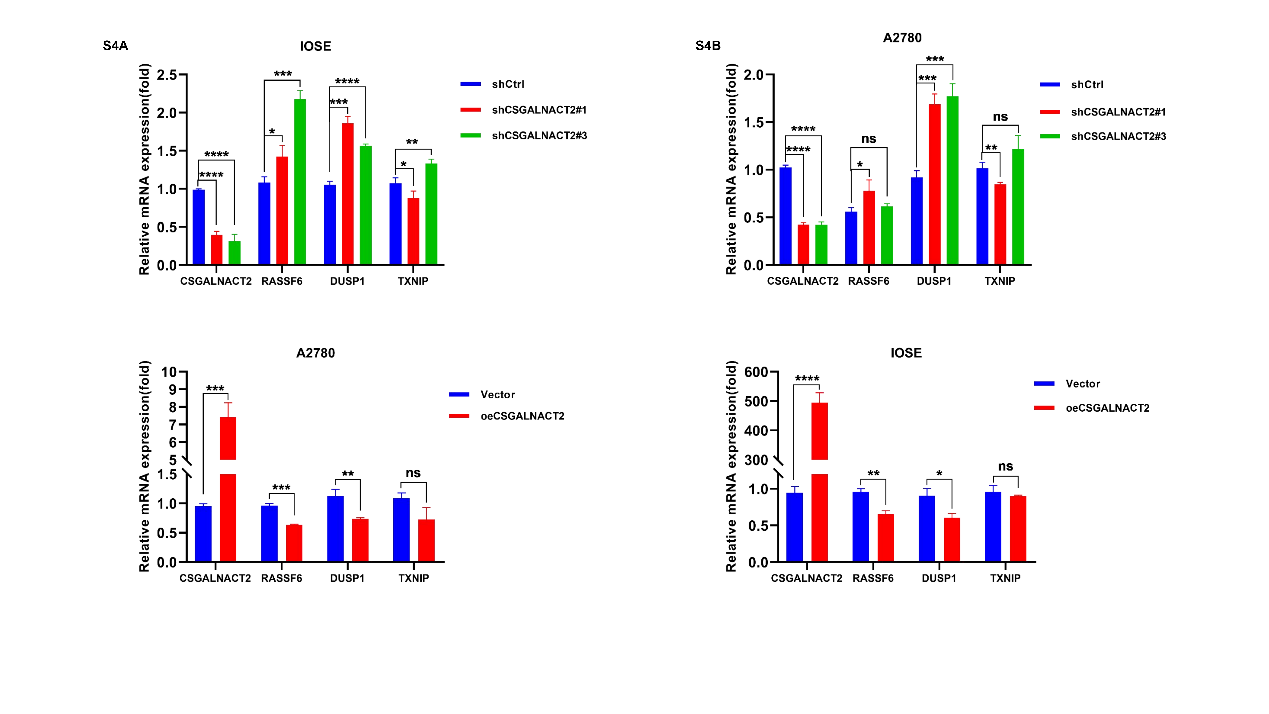


**Supplementary** **Fig. 4**. **A-B** The expression of core genes, such as RASSF6, DUSP1, and TXNIP in A2780 cells **A** and IOSE cells **B** with up- and down-regulated CSGALNACT2 by qRT-PCR.

**
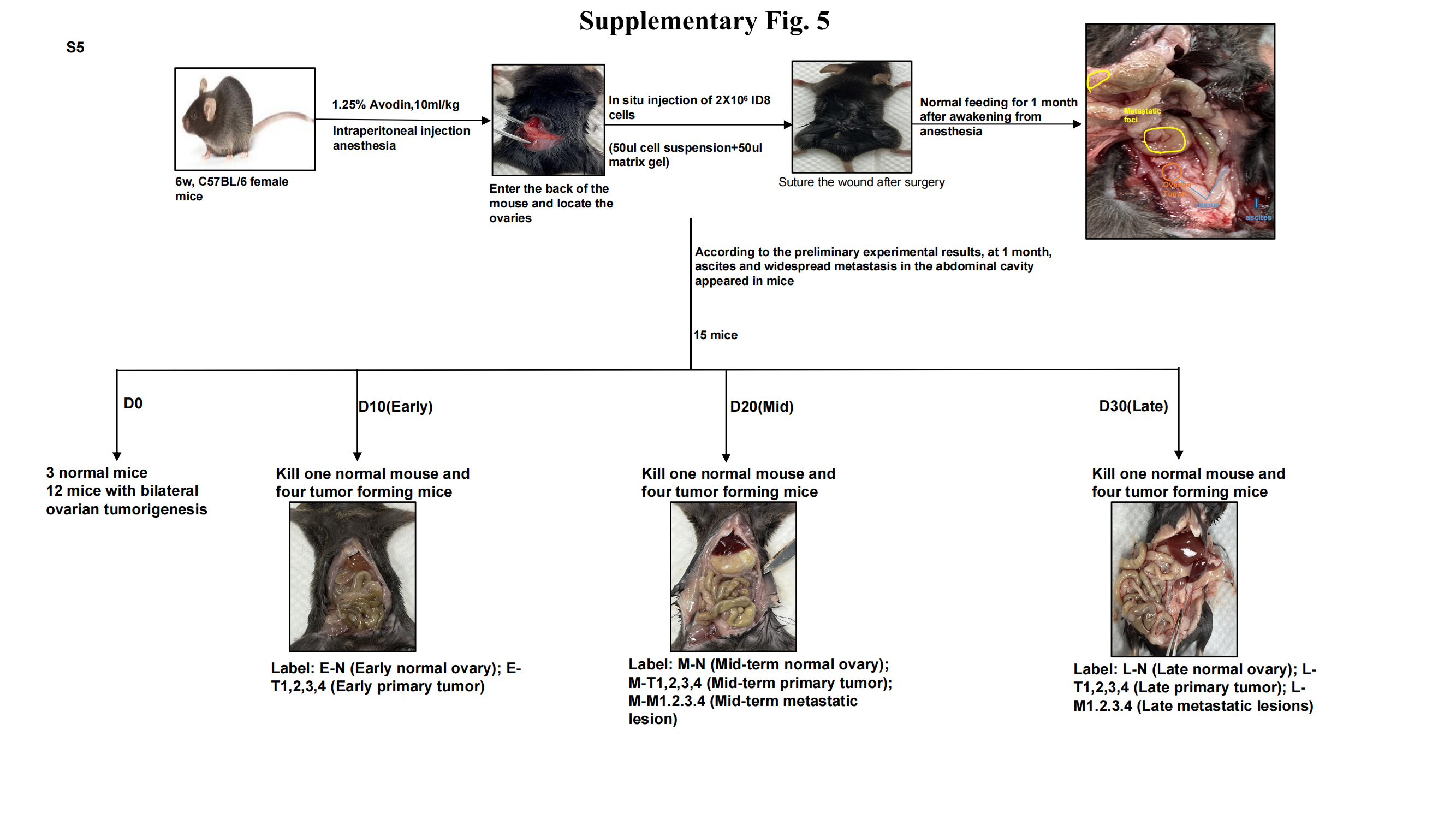
Supplementary** **Fig. 5**. Technical roadmap for the design of animal experiments.


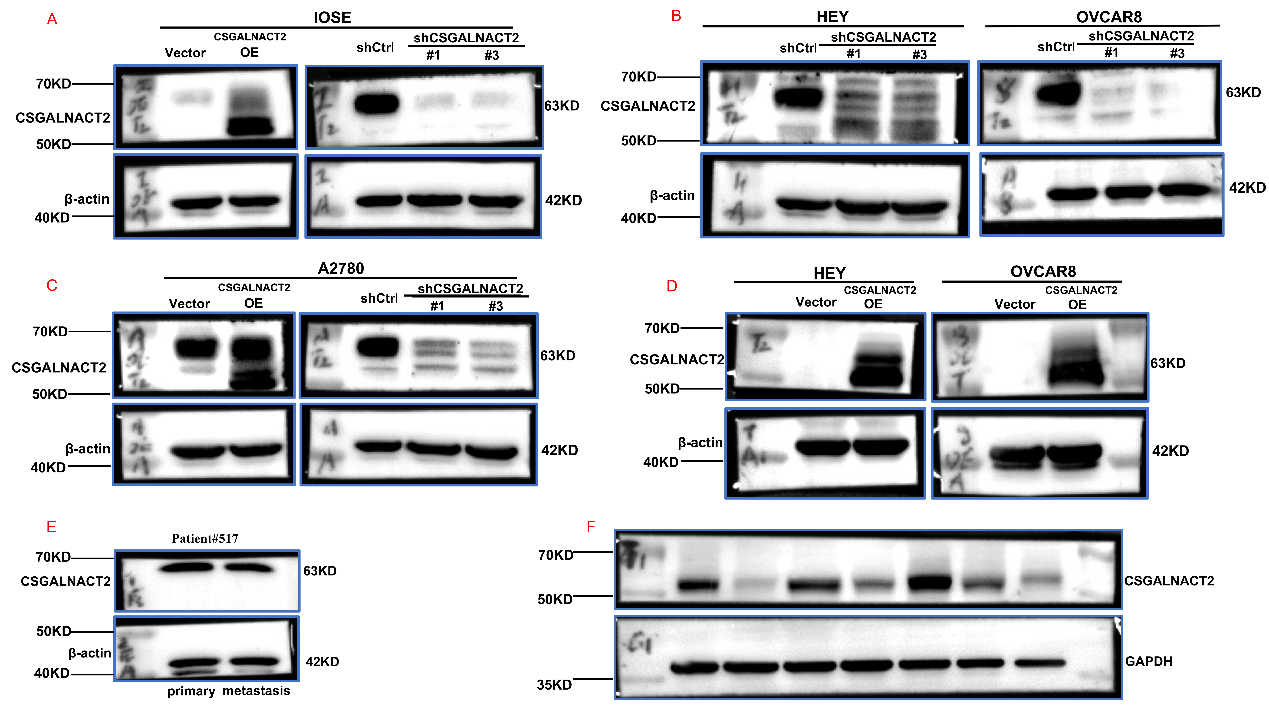


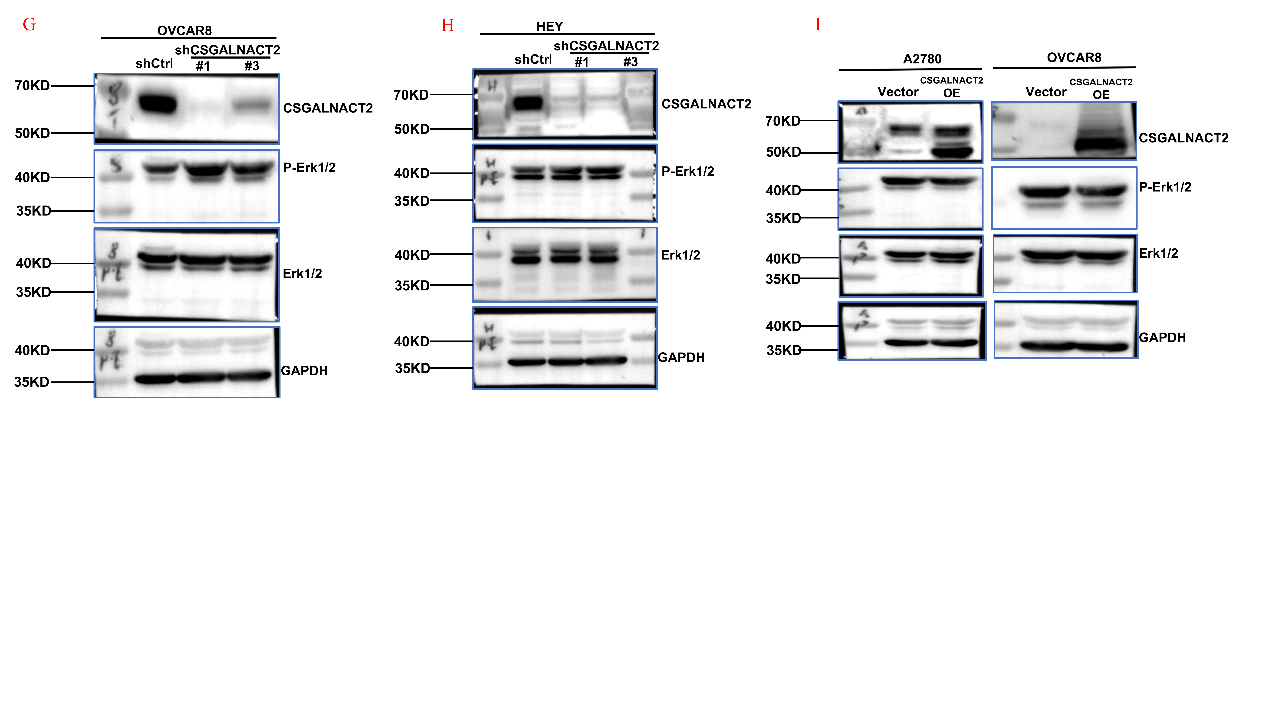


**Supplementary** **Fig. 6**. Western Blots ORIGINAL figures.
